# Characterizing landscape configuration effects on eastern spruce budworm infestation dynamics

**DOI:** 10.1007/s10980-025-02203-z

**Published:** 2025-08-27

**Authors:** Tommaso Trotto, Nicholas C. Coops, Alexis Achim, Sarah E. Gergel, Dominik Roeser

**Affiliations:** 1 Department of Forest Resources Management, University of British Columbia, 2424 Main Mall, Vancouver, BC V6T 1Z4 Canada; 2 Département des sciences du bois et de la forêt, Université Laval, 2425 rue de la Terrasse, Québec, QC G1V 0A6 Canada; 3 Department of Forest and Conservation Sciences, Faculty of Forestry, University of British Columbia, 2424 Main Mall, Vancouver, BC V6T 1Z4 Canada

**Keywords:** Boreal, Non-stand replacing disturbances, Landsat, Probabilities, Time series

## Abstract

**Context:**

Spruce budworm (*Choristoneura fumiferana*, Clem, SBW) is the largest defoliator of boreal and mixedwood forests in North America. Its impact is directly linked to the quality and availability of primary host species such as balsam fir (*Abies balsamea*, (L.) Mill.) at the stand level. At the landscape level, the ability of SBW to disperse over long distances suggests that the configuration of available resources may also play an important role in affecting infestation success and outcomes.

**Objectives:**

We hypothesized that fragmented landscapes characterized by smaller and more dispersed conifer patches intermixed with other land cover types may promote infestations thanks to the ability of SBW to saturate the landscape and avidly consume limited resources. To test our hypothesis, we investigated to what degree landscape configuration plays a significant role in modulating defoliations using spectrally-segmented forest stands. Next, we determined the major drivers of infestation probabilities and how they have evolved as a result of landscape configuration changes.

**Methods:**

We capitalized on a combination of Landsat imagery, Forest Resource Inventory (FRI) data, and probability estimates from random forest models to investigate multi-scale effects of landscape configuration on SBW infestations over 13 years in eastern Quebec, Canada, in a spatially-explicit fashion. Based on annual best available pixel composites of surface reflectance derived from Landsat, we superimposed a 400 m tessellation over which we extracted six landscape configuration metrics describing area, aggregation, and shape for infested and non-infested conifer forest patches. Next, probability estimates from two sets of random forest models were extracted from the configuration metrics at annual time steps and for the entire length of the time series.

**Results:**

Landscapes characterized by greater fragmentation of conifer patches had a higher risk of infestation. In such landscapes, greater fragmentation was indicated by smaller and more variable-sized conifer patches, with a mean patch area < 40 ha (CV > 100 ha) and a landscape patch index < 50 %. In addition, such areas had more isolated patches and more complex shapes, as indicated by cohesion < 97 %, landscape shape index > 3, and shape > 1.35. The landscape patch index, quantifying the percentage of landscape covered by the largest coniferous patch, had the greatest influence on SBW infestations. These results confirmed our initial hypothesis that a higher level of fragmentation of conifer patches may favor infestation establishment.

**Conclusions:**

We demonstrated the use of freely available Landsat imagery to extract configuration metrics in a spatially-explicit fashion. Further, we highlighted the value of using probability estimates to capture landscape configurations at higher risk of infestations. This knowledge can inform forest management practices, such as where harvesting operations may be carried out on the landscape or where planting may be prioritized to reduce conifer stand fragmentation and infestation risk.

**Supplementary Information:**

The online version contains supplementary material available at 10.1007/s10980-025-02203-z.

## Introduction

The understanding and monitoring of forest disturbances is crucial to inform management practices aimed at maintaining or improving forest health (Trumbore et al. 2015) in the face of climatic changes and their influence on forest dynamics (Dale et al. 2001; Seidl et al. 2017; Pureswaran et al. 2018; Altman et al. 2024). In North America, the increased presence of wildfires and non-stand replacing disturbances such as insect outbreaks has disturbed extensive areas in recent years (Gauthier et al. 2014), with potentially major ecological and economic implications (Candau & Fleming 2005). From a Canadian perspective, the abundance and distribution of eastern spruce budworm (*Christoneura fumeraria*, Clem, SBW), a native defoliator of spruce and fir forests, is related to changes in landscape-scale bioclimatic conditions (Candau & Fleming 2005; Pureswaran et al. 2018), which in turn modulate plant phenology, as well as host distribution and connectivity. SBW preferentially feeds on balsam fir (*Abies balsamea* L. (Mill.)), yet may extend to secondary hosts such as white spruce (*Picea glauca* (Moench) Voss) and black spruce (*Picea mariana* Mill.) under severe infestations (Nealis & Régnière 2004; Pothier et al. 2012; Bognounou et al. 2017);. The risk of endemic populations escalating to epidemic levels is also attributed to landscape saturation mechanisms. Such mechanisms include long-distance dispersal of SBW adults to areas where SBW is already endemic, coupled with high larval survival rates, thereby effectively increasing SBW densities (Régnière & Nealis 2007, 2019; Régnière et al. 2013). Historically, cyclic infestations occur at 30-40 year intervals and tend to result in patches of severely infested trees in a matrix of lighter infestations (Bouchard & Pothier 2010). Repeated attacks increase tree- and stand-level mortality, reduce vigour by limiting photosynthetic capacity (Candau & Fleming 2005), and alter species composition (Bouchard et al. 2006) and structure with implications on canopy cover and tree height (Trotto et al. 2024).

The movement of SBW adults through the landscape is affected by forest composition. A greater proportion of host tree species can allow endemic SBW populations to escalate to epidemic levels, resulting in more widespread and extensive infestations (Régnière & Nealis 2007; Sturtevant et al. 2015; Kneeshaw et al. 2021; Cooke et al. 2024). In contrast, a more heterogeneous composition of tree species, such as a greater proportion of mixed and hardwood forests alongside fir and spruce, may reduce the severity of infestations (Zhang et al. 2018, 2020). Composition, which refers to the proportions of different tree species in a stand or landscape, does not consider the spatial organization of stands. This spatial organization is important to consider for highly mobile organisms like SBW (Sturtevant et al. 2013). Greater landscape connectivity, greater chances of finding suitable host species, as well as higher stochasticity of encounters with natural enemies, have the potential to affect the outcome of an infestation (Peltonen et al. 2002; Johnson et al. 2004; Lindenmayer et al. 2009). For example, interspersion of hardwood or mixedwood stands among fir and spruce patches, resulting in decreased conifer connectivity, may affect the likelihood of populations to infest host trees, potentially reducing infestation spread and success (Zhang et al. 2020). Similarly, smaller patches of host trees may favor crowding of endemic SBW populations (Royama 1984; Yamamura 2002). Therefore, forest configuration plays a role in affecting the outcomes of an infestation and its movement across the landscape.

Studies conducted in the southern border of SBW habitat range in Ontario, Canada, and Minnesota, US have revealed varying effects of forest configuration on the severity of defoliation, frequency of infestation cycles, and synchronism of attacks. Robert et al. (2018) observed that infestations manifested with lower severity, higher frequency of outbreak cycles, and lesser spatial synchronism of infestations under greater landscape fragmentation and in younger forests with a lower component of host species. In particular, greater fragmentation was expected to result in lower SBW dispersal success (Nealis 2016) and greater control by natural enemies (Royama 1984). In southern Quebec, Canada, McNie et al. (2023) showed that the risk of infestation onset increased in more fragmented landscapes. In particular, they assessed landscape configuration based on the landscape shape index (LSI), a configuration metric that describes landscape complexity as a function of patch shape and landscape size (McGarigal et al. 2023) and can be used as a proxy for landscape fragmentation. They showed how greater landscape fragmentation, expressed as a 10% increase in LSI, increased the risk of infestation onset between 26.4% and 36.5%. However, current research investigating landscape configuration effects on SBW infestation dynamics is limited to landscape-level analyses conducted at a single point in time or lacks the spatially-explicit nature suitable to study SBW infestation dynamics at the level of neighborhood or stands or their evolution.

Remote sensing technologies have facilitated spatial, spectral, and temporal analyses of insect disturbances at varying scales (MacLean et al. 2001; Trumbore et al. 2015; Senf et al. 2017a, b). Numerous studies have investigated satellite remote sensing for the assessment of SBW infestations, thanks to the availability of long time series and spatially-explicit data that can capture the spatio-temporal variability of insect disturbances (Senf et al. 2017a, b; Rahimzadeh-Bajgiran et al. 2018; Coops et al. 2020; Donovan et al. 2021). Despite the breadth of research on SBW using remote sensing, to our knowledge, no study has yet incorporated landscape configuration in the characterization of infestations at the landscape level in a spatially-explicit fashion. One challenge that arises is the lack of temporally-consistent and spatially-objective segmentation of forest stands. However, the availability of long time series of freely available satellite imagery from Landsat and well-established object-based segmentation algorithms permits meeting this information need by building wall-to-wall, spatially-consistent segments representing forest stands based on spectral values and contextual information, for example related to disturbance history (Wulder et al. 2024). Moreover, satellite-based stand segmentation has the critical advantage of being agnostic to management type or land status (Wulder et al. 2024). Following segmentation, spatial relations and organization of forest stands can be measured using a set of stand-level configuration metrics that are easily interpretable. Furthermore, configuration changes can be tracked over time to understand the spatio-temporal evolution of infestations in response to a changing landscape. By studying how close some configurations resemble those that were infested in the past, it would be possible to estimate the likelihood of present configurations to experience SBW infestations. Such likelihood would describe to what degree a specific landscape configuration matches a known configuration that had experienced defoliations in the past, rather than describing its potential to be infested in the future. This information is valuable in that managers can make informed decisions regarding what parts of the landscape are at a higher risk of infestations and actively manage them to reduce the risk, for example by altering their shape or size (Johns et al. 2019; MacLean et al. 2019).

In this paper, we aim to leverage freely available Landsat imagery to estimate (i) annual infestation probabilities over southern Quebec and (ii) the relative influence of landscape configuration in modulating infestation severity over 13 years. To our knowledge, this represents a first attempt to characterize the temporal evolution of SBW infestation probability based on landscape configuration in a spatially-explicit fashion. In doing so, two key questions will be addressed. First, we will investigate how different landscape configurations, derived from spectrally-segmented forest stands, affect the likelihood of severe or moderate SBW infestations and identify which configurations play the most important role in reducing this likelihood. Secondly, we will examine how the likelihood of severe or moderate SBW infestations changes over time.

## Study area and data

### Study area description

This research was conducted in Lac Saint-Jean, Quebec, Canada (LSJ), a boreal mixedwood region covering approximately 20.355 km^2^. LSJ is host of two bioclimatic domains. The Balsam Fir—Yellow Birch (*Betula alleghaniensis* Britt.) and the Black Spruce—Feathermoss domain. The first extends across the southern part of the region and is dominated by a mixture of balsam fir, yellow birch, eastern cedar (*Thuja occidentalis* L*.*), and white spruce. The latter is represented by an assemblage of black spruce and balsam fir, along with trembling aspen (*Populus tremuloides* Michx.) and white birch (*Betula papyrifera* Marshall), and covers the northernmost part of the region. The physiography of LSJ is relatively flat, with highlands ranging between 60 m and 215 m above the southern lowlands (Clibbon & Bergeron 1962). Climatic projections using ClimateNA (Wang et al. 2016) show a likely increase by 23% in precipitation and an increase of 5.9 °C and 4.7 °C for minimum and maximum temperature by the end of the century. These values are an average across four shared socioeconomic pathway scenarios of a 13 general circulation model ensemble (Wotherspoon et al. 2022).

### Data sources

In this analysis, we capitalized on the strength of two key datasets. First, we utilized annual Landsat spectral composites over the region to derive fine-resolution stand characteristics indicative of a landscape configuration prior to and during the infestation. We then linked these to the Forest Resource Inventory (FRI) from the Quebec Ministère des Ressources naturelles et des Forêts. The FRI provides forest health sketch maps, which are available annually and are produced by experienced forest health professionals viewing the forest from above as part of regular forest health assessments in the region. The Landsat imagery, with its high spatial resolution and geometric accuracy, provides an accurate, spatially-explicit, reference to map stand characteristics. Conversely, the forest health information is less well spatially registered, human-interpreted, and is variable in scale. Although, it provides critical information on classes of SBW infestation derived from trained professionals, as the infestation moves over the landscape.

#### Landsat spectral composites

To derive annual mosaics of landscape configuration, we used Landsat best-available-pixel (BAP) image composites available through the National Terrestrial Ecosystem Monitoring System (NTEMS) and generated based on the Composite2Change approach. This approach produces gap-free, annual Landsat surface reflectance BAP image composites (White et al. 2014; Hermosilla et al. 2015). We downloaded layers from 2008 to 2020 at 30 m resolution. We also utilized land cover classification maps produced with the Virtual Land Cover Engine (Hermosilla et al. 2022b), and disturbance layers reporting fire and forest harvesting occurrences to create a no data mask (Hermosilla et al. 2015, 2016).

#### FRI disturbance mapping

We utilized annual aerial overview surveys (AOS) conducted by the Quebec Ministère des Ressources naturelles et des Forêts with a minimum mapping unit (MMU) of 0.1ha. These surveys are conducted by manual delineation of current defoliation into three different severity classes (light, moderate, severe) depending on the portion and amount of infested foliage (Ministère des Ressources Naturelles et des Forêts 2024). Light infestations indicate loss of foliage in the upper third canopy of a few trees. Moderate infestations indicate loss of foliage in the upper half canopy of most trees. Severe infestations indicate loss of foliage across the crown length of most trees. In addition, we used annual vector layers of forest harvesting operations with a 0.1ha MMU for masking purposes. The first available recent occurrence of SBW for the study area is dated 2008.

## Methods

**Fig. 1:**
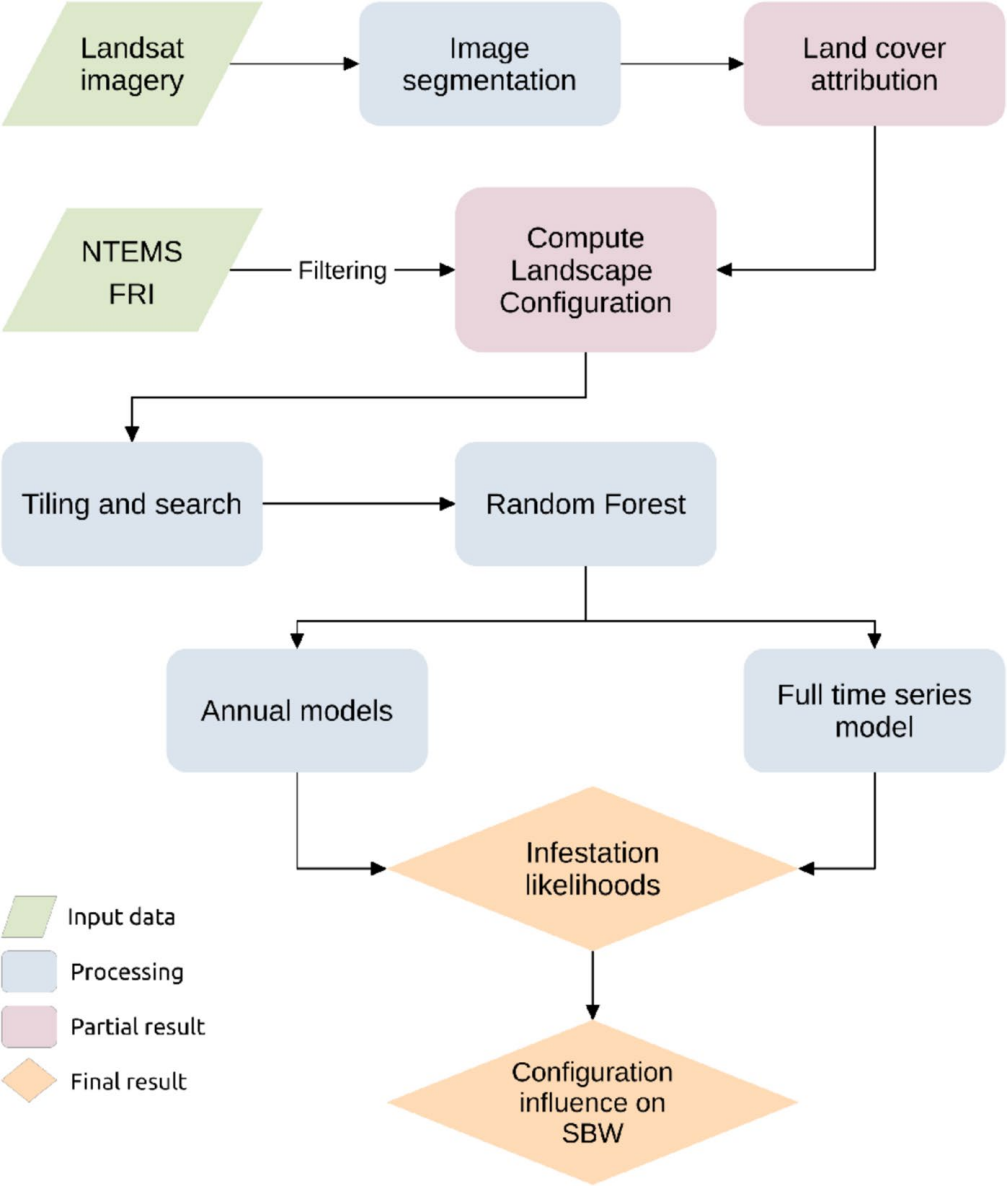
Illustration of the workflow used in this research

The methods applied in this paper are described in Figure 1 and detailed in the following sections.

### Data compilation

For each year, the available AOS disturbance layer describing SBW infestation severity was rasterized at 30 m to match the Landsat products, and an integer class (1- 3) was assigned to the corresponding severity levels. Fire, harvesting, roads, and water bodies were removed from the analysis, allowing us to isolate the effect of SBW on landscape configuration from other factors. In particular, fires were removed annually between 2008 and 2020, whereas harvesting was removed annually for the same period and up to 15 years prior to the beginning of the time series in 2008. Undisturbed areas were identified annually as pixels that did not experience any disturbance up to 15 years prior to 2008, thus including fires, harvesting, and SBW defoliations. To provide some level of generalization, a 1 ha MMU was used for the undisturbed dataset.

### Stand segmentation

To produce temporally-consistent segments representing forest stands to study the evolution of landscape configuration and its potential role in affecting SBW infestation likelihood, a multiresolution segmentation algorithm available from the Object Analyst module in Catalyst (PCI Geomatics, Canada) was run on the BAP composites for each year between 2008 and 2020. We followed the same methodology as Wulder et al. (2024). This is an object-based segmentation routine (Blaschke 2010) that incorporates spectral, spatial, geometric, and contextual information. To generate relevant segments for forest inventory purposes, we input the Landsat composites containing red, near infrared (NIR), and shortwave infrared (SWIR) band information from BAP composites, as well as the no data mask. The use of red, NIR, and SWIR bands allowed us to capture different stand characteristics associated with coniferous forest cover. In particular, changes in red and NIR are linked to changes in pigments and forest structure (Key and Benson, 2006), whereas changes in SWIR can be caused by changes in foliage water content (Wang et al. 2008). For instance, a stand attacked by SBW may result in an increase of SWIR and red bands over time due to a reduction in foliage water content and vegetation browning as larvae feed on current and previous year foliage. Conversely, severely infested stands characterized by high tree mortality may result in a decrease in NIR due to a loss in standing vegetation and greater soil exposure.

In our stand segmentation, we maintained the same segmentation parameters as Wulder et al. (2024) (scale = 50, shape = 0.3, compactness = 0.5). Stands smaller than 0.45 ha were removed if isolated or aggregated into the neighboring stand with the longest boundary (Wulder et al. 2024). Once the segmentation was completed, each segment was assigned the modal land cover type based on the land cover maps (Hermosilla et al. 2022b).

### Measuring landscape configuration

To provide spatially-explicit information on the landscape configuration evolution, we implemented a tiling system for each segmented BAP composite (2008—2020). Landscape metrics provide summary statistics for a given landscape. To enable the characterization of spatial patterns at a finer resolution, we superimposed a 400 x 400 m (16 ha) tessellation across the study area, effectively creating a unique landscape for each tile. This allowed us to characterize the spatial patterns of each focal landscape and its neighboring landscapes. Within each tile, a set of six landscape metrics was computed at the class- (4) and landscape-level (2) using an 8-neighboring rule (Table 1). Class-level metrics describe inter-class relations and within-class summaries, while landscape-level metrics describe general characteristics of the entire landscape. We selected complementary metrics that allowed us to describe different aspects of the landscape (Gergel 2007; Turner and Gardner 2015), including size and aggregation (e.g. LSI), as well as shape and complexity (e.g. SHAPE) at different levels of organization. LSI is computed from patch edges, yet it is used as an aggregation metric because it effectively captures actual edge length in relation to a hypothetical scenario consisting of fully-aggregated patches of the same class (McGarigal et al. 2023).

**Table 1:** Summary of landscape metrics used in this study. AREA_CV and SHAPE are the only metrics computed at the landscape level

**Metric (abbreviation)**	**Type**	**Description**
Class area (PA)	A	Mean patch area by class in hectares.
Cohesion (COHESION)	A	Percentage of patch connectedness by class. Higher values indicate higher connectedness.
Largest patch index (LPI)	A	Percentage of land covered by the largest patch of each class.
Landscape shape index (LSI)	A	Shape complexity by class measured as shape departure from a square. Higher values imply more complex or spatially-distinct patches.
Coefficient of variation of landscape area (AREA_CV)	A	Variability in patch area in the landscape in hectares.
Shape index (SHAPE)	S	Mean ratio between patch perimeter and square root of patch area computed for the entire landscape.

Type indicates the metric type. *A* area and aggregation, *S* shape. Metrics definition can be found in McGarigal et al. (2023)

To assess the effect of landscape configuration on SBW infestation likelihood, we computed landscape configuration for disturbed and undisturbed tiles based on the AOS disturbance data and exclusively for conifer stands, which represented the large majority of forested land in our study area. In particular, a tile was considered disturbed if at least 50% of the total tile area was affected by a severe or moderate infestation in that year. Undisturbed tiles were attributed if the total disturbed area per tile was < 25% or if no disturbance, regardless of the type, occurred for the length of the study period, and up to 15 years prior. Tiles with disturbed areas between 25 and 50% were removed (~ 10%) to focus only on tiles characterized by larger disturbances and stronger landscape configuration responses. We limited the use of tiles to only those whose modal land cover was conifers in each year, as derived from NTEMS. This was done to avoid tiles being marked as undisturbed simply because of the absence of host species. Ultimately, this resulted in a dataset of configuration metrics and corresponding binary class outcomes (disturbed/undisturbed).

### Estimate infestation likelihoods

To produce spatially-explicit, tile-level probability estimates of infestation onset, we utilized a random forest classifier (Breiman 2001). The model incorporated information from each focal tile alongside contextual information derived from surrounding tiles at multiple scales. This step allowed us to augment the training sample, reduce edge artifacts, and force attention to the relationships with neighboring tiles, which is important for highly mobile organisms like SBW. First, we computed landscape configuration metrics for each focal tile between 2008 and 2020. Next, we defined a region of interest around each tile to incorporate the 3 × 3 (144 ha) and 5 × 5 (400 ha) surrounding tiles, and computed configuration metrics on those larger regions for the same period. Configuration metrics for the focal tile received a weight of 1, the 3 × 3 region received a weight of 0.4, while the larger 5 × 5 region received a weight of 0.2. The resulting dataset was balanced to retain the same number of disturbed and undisturbed tiles, for each year, as well as centered to the median and scaled to the 5 - 95 interquartile range.

Tiles were classified annually as either disturbed or undisturbed based on the AOS surveys for that year. For model generation, only tiles within a user-defined search radius for each year were input into the model. This step was taken (i) recognizing that the spread of moderate or severe SBW infestations is limited on an annual basis over the landscape, (ii) to reduce the effect of a latitudinal species composition gradient given the extensive north-south orientation of our study area (i.e. greater proportion of host species in the south than in the north), and (iii) contrast neighboring disturbed and undisturbed stands to investigate preferential landscape configurations for outbreak movement. To do so, for each year we first determined the geometrical centroid of the disturbance polygons from the FRI and built a buffer around it to encompass the northernmost polygon with an area of at least 5 ha, plus a 75 km radius extension. The radius extension represents a median dispersal range distance for migratory SBW adults (Greenbank et al. 1980; Sturtevant et al., 2013).

Two sets of models were generated. First, we generated a full time series model that included configuration metrics for disturbed and undisturbed tiles for all 13 years of data. Model parameters were number of decision trees set to 100, minimum sample size per terminal leaf set to 9, maximum depth set to 10, all available features to determine the best split, or default values to reduce overfitting. Second, we fit 13 individual annual models with the same parameters where only tiles that were infested or undisturbed within our search radius were used for the specific year. For each model, we estimated the infestation probabilities for disturbed and undisturbed tiles and used them to assess model performance. To do that, we thresholded the class probabilities into binary categories to match the binary class outcomes (disturbed/undisturbed). The assessment was conducted using two cross-validation (CV) approaches. A time series CV routine for the full time series model and a stratified 5-fold CV for the annual models. Thereby, the full time series model was trained using all data available up to and excluding the processed year, and tested on the held-out. For example, the model for year 2020 was trained using all data between 2008 and 2019 and tested on the 2020 dataset. This is similar to a K-fold cross validation, although it is necessary because it accounts for the temporal autocorrelation of the data. During each fold, we computed the accuracy score, Receiver Operator Characteristic (ROC) curve and corresponding Area Under the Curve (AUC) and threshold. The threshold represented the appropriate cut-off value of a continuous dataset in a binary classification task (Habibzadeh et al. 2016). This value was selected as the point along the ROC curve that maximized the difference between true positive rate and false positive rate (Manel et al. 2001). True positive and false negative rates represent the rates at which probability values correspond to truly infested tiles as per the AOS surveys. The overall model performance was evaluated by computing the AUC from all estimated class probabilities from each fold (Fawcett 2006). AUC values greater than 0.5 indicate attribution better than random chance for binary classification tasks.

From both random forest models, we extracted the partial dependence of each configuration metric and compared them. Thereby, we were able to determine whether annual model predictions were consistent with the full time series model. Finally, to assess the importance of each landscape configuration on the estimation of infestation likelihoods, we extracted the distribution of feature importance from each annual model based on 100 model permutations. Permutation feature importance is a technique whereby the degradation of the model’s accuracy score can be observed by randomly shuffling the values of the input features, thus offering insights into the importance of each variable in the model’s prediction (Breiman 2001).

## Results

The total cumulative area affected by SBW for all years combined between 2008 and 2020 amounts to approximately 2.9 Mha. Until 2015—2016, the area covered by reported infestations was limited to the southernmost part of LSJ, with infestations moving northward in more recent years. By 2020, approximately 44% of our study area was under some form of infestation, with a majority of low and moderate severity infestations. Northern regions had not been subject to SBW attacks according to the AOS surveys in the same period.

### Characteristics of segmented stands

**Fig. 2:**
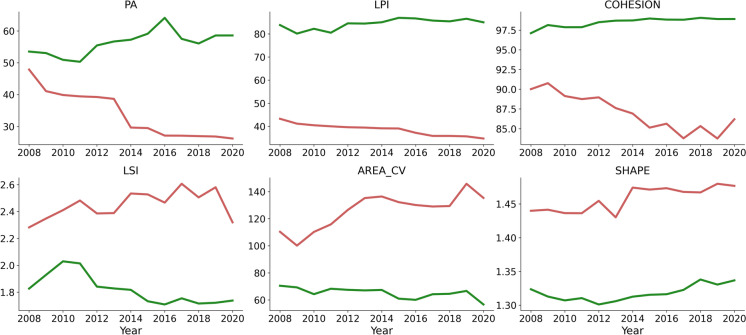
Temporal changes of landscape configuration between 2008 and 2020. The red line represents the averaged configurations measured in disturbed tiles, while the green line represents the averaged undisturbed conditions, for each year. *PA* patch area, *LPI* landscape patch index (landscape percentage covered by the largest patch), *COHESION* cohesion (patch connectedness), *LSI* landscape shape index (shape complexity of a patch), *AREA_CV *coefficient of variation of landscape area within a tile, *SHAPE* shape (patch shape). For the full interpretation of each configuration metric, the reader is referred to Table 1

Stand segmentation following the aggregation of small segments resulted in an average stand size of 13.5 ± 25.2 ha across all years. Segment areas remained stable from 2008 (average 13.6 ± 25.2 ha) to 2020 (average 13.3 ± 24.9 ha). Coniferous land cover was the most dominant and represented 70.5% of the total land in 2008 and stably decreased to 64.5% by 2020, followed by mixedwood lands, which covered 8.7% in 2008 and stably increased to 12.4% by 2020. Most of the declined coniferous land cover converted into barren land or shrubs as a result of harvesting, or transitioned into mixedwood land cover. Defoliation at different severity levels occurred on the same stands over time. In total, 52% of the stands were reported to have experienced low infestation severity, 40% moderate infestations, and 21% severe infestations. Undisturbed areas in 2008 accounted for approximately 54% of the total area and reduced to about 24% by 2020. Landscape configuration of coniferous stands within disturbed and undisturbed tiles changed over time (Figure 2, Figure S1). The northward expansion of SBW resulted in increased levels of fragmentation characterized by smaller and more dispersed conifer patches (lower PA, LPI, and COHESION) starting in 2008. Shape complexity of conifer patches increased over time, as represented by an increase in LSI and SHAPE. However, LSI showed more variability than SHAPE in later years as infestations became more severe. Similarly, the variability in patch area at the landscape level (AREA_CV) increased as infestation progressed.

### Full time series model

**Fig. 3:**
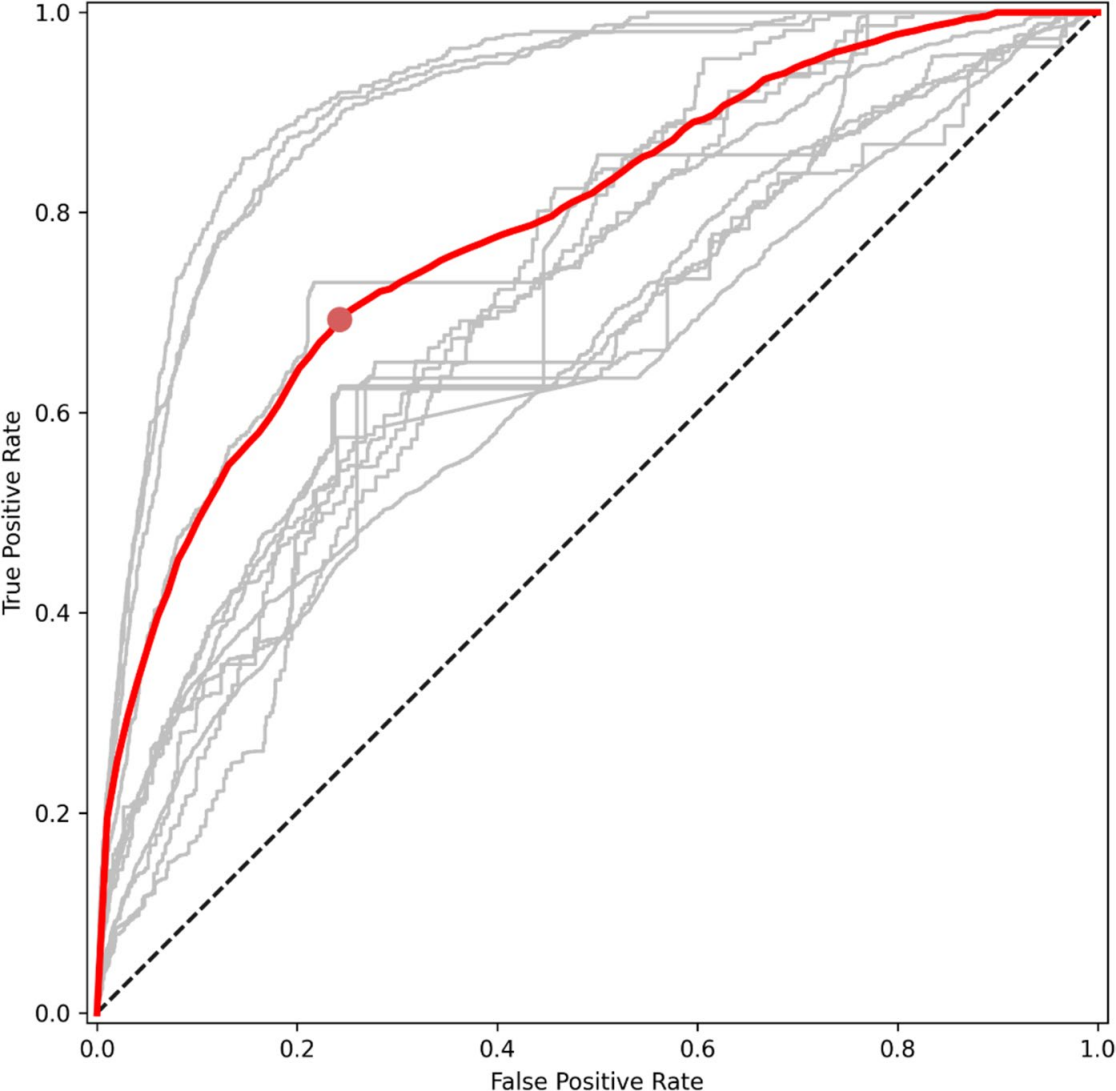
Receiver-Operator Characteristic (ROC) curve for the random forest model. In grey, averaged ROC curves for each year. In red, ROC curve obtained from the full time series model. The red dot represents the optimal threshold value as the maximum absolute difference between true positive rates and false positive rates

**Fig. 4:**
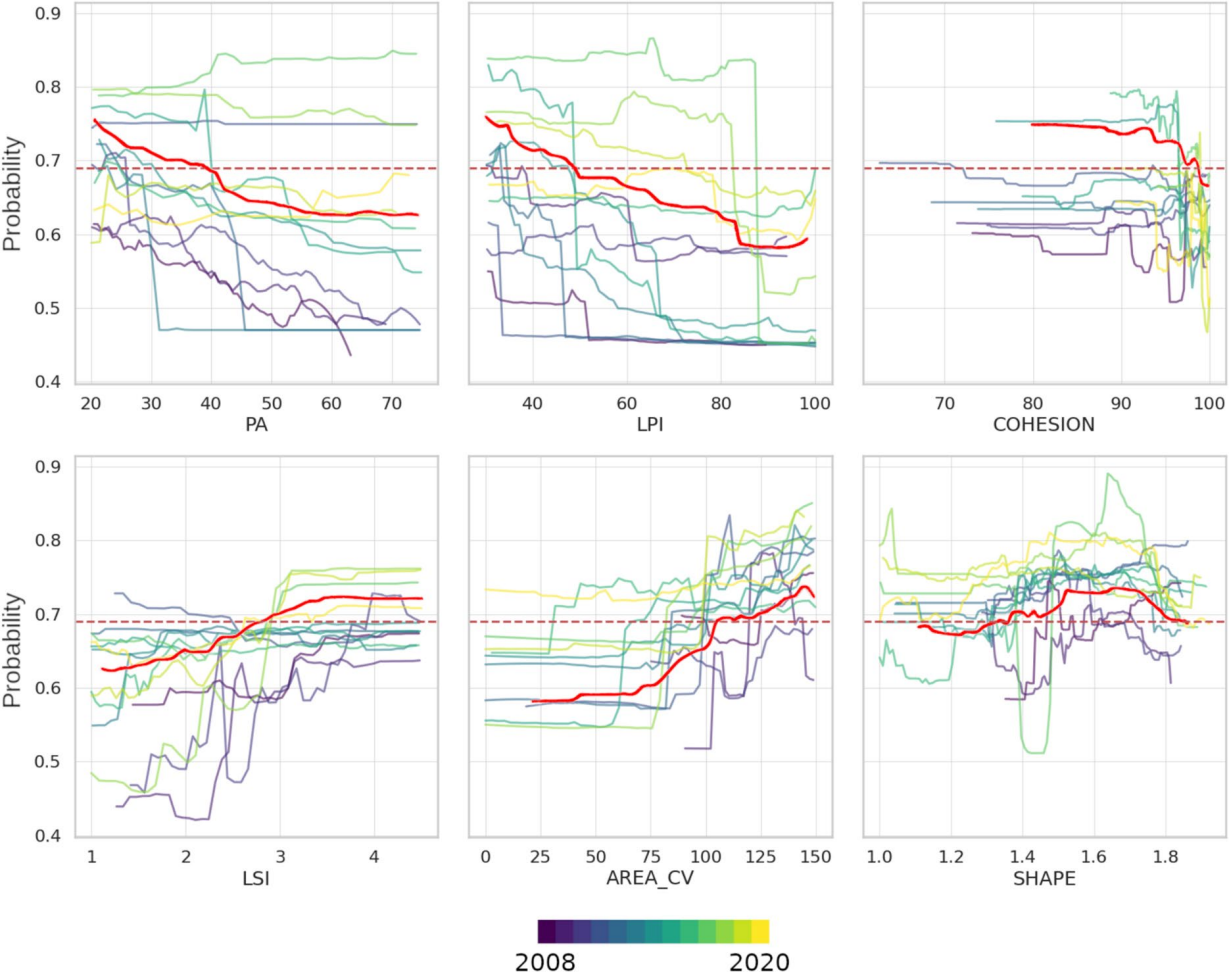
Partial dependence display for each annual random forest model colored by year. The solid and dashed red lines represent the partial dependence of each configuration metric based on the full time series model and its corresponding AUC threshold, respectively. *PA* patch area, *LPI* landscape patch index (landscape percentage covered by the largest patch), *COHESION* cohesion (patch connectedness), *LSI* landscape shape index (shape complexity of a patch), AREA_CV coefficient of variation of landscape area within a tile, SHAPE shape (patch shape). For the full interpretation of each configuration metric, the reader is referred to Table 1

The AUC for the full time series model was 0.72, with an optional threshold value of 0.69 (Figure 3). Probability values higher than this threshold were considered as representative of severe or moderate infestations at the tile level. Partial dependency plots showed that higher infestation likelihoods were associated with higher fragmentation, represented by smaller conifer patches of variable area and more complex shape (PA < 40 ha, LPI < 50 %, COHESION < 97 %, LSI > 3, AREA_CV > 100 ha, and SHAPE > 1.35) (Figure 4). We observed an overall linear response of PA and LPI, where larger patches were associated with a lower infestation probability. Opposite trends were observed for the coefficient of variation in patch area (AREA_CV). Highly connected patches (COHESION) resulted in abrupt reductions in infestation likelihood, while lower values consistently resulted in probabilities above the threshold. LSI had comparatively lower influence on the model than any other metrics, yet greater shape complexity resulted in higher infestation likelihood. The influence of patch shape (SHAPE) on the model was non-linear, with higher probability values corresponding to SHAPE between 1.35 and 1.8, and a subsequent decrease as patches became more complex.

### Annual models

The lowest model performance was recorded in 2008 (0.67) and peaked between 2011 and 2013 (~0.90). From 2014 to 2019, model performance fluctuated between approximately 0.65 and 0.75. True positive rates fluctuated over time, with higher values (> 0.65) in later years since 2016, while false positive rates stayed lower than 0.35, except for 2008—2010 with values between 0.33 and 0.45. Accuracy scores based on the FRI labels ranged between 0.58 and 0.85 across the time period, with differentiation between disturbed and undisturbed areas being worse in early years compared to later years. Between 2008 and 2010, accuracy scores ranged between 0.58 and 0.61, and increased from 2011 onward between 0.63 and 0.85.

Patch shape and coefficient of variability of patch area (LSI and AREA_CV) had the clearest influence on the model predictions and did not fluctuate as strongly as other metrics, on an annual basis (Figure 4). We did not observe a clear trend in patch area and LPI on an annual basis, although an increase in patch area and LPI was associated with a reduction in infestation likelihoods. Greater conifer patch cohesion did not result in an increase in infestation likelihood until later years, when less aggregated patches resulted in an abrupt reduction in infestation likelihood, similarly to the full time series model. SHAPE showed no clear response based on the individual annual models, although a general increase in infestation probabilities was associated with intermediate shape complexity values.

### Feature importance

**Fig. 5:**
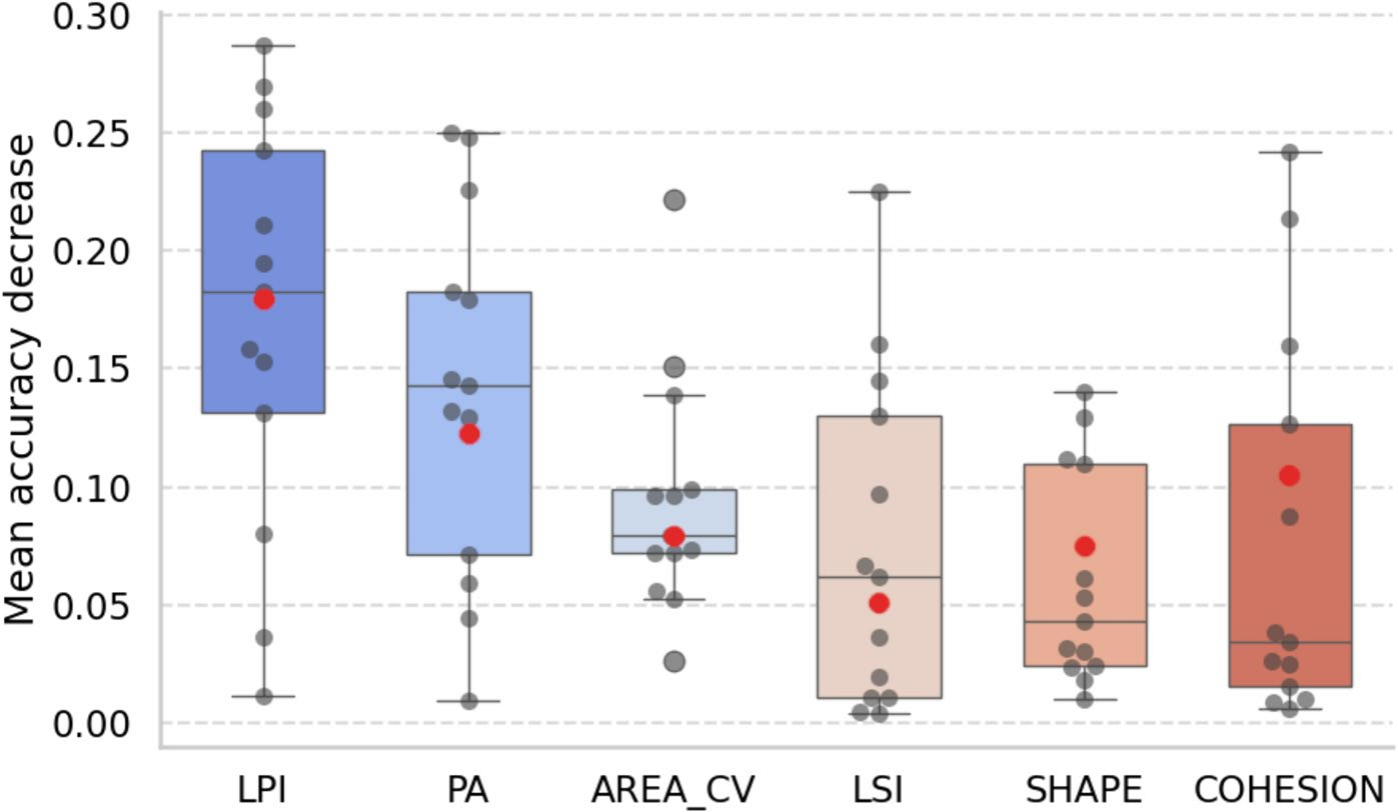
Boxplot of variable importance distribution for each configuration metric. Distributions are calculated from the annual random forest models. The red dot represents the mean accuracy decrease based on the full time series model. Boxes are sorted by descending median values. *PA* patch area, *LPI* landscape patch index (landscape percentage covered by the largest patch), *COHESION* cohesion (patch connectedness), *LSI* landscape shape index (shape complexity of a patch), *AREA_CV* coefficient of variation of landscape area within a tile, *SHAPE* shape (patch shape) . For the full interpretation of each configuration metric, the reader is referred to Table 1

For both models, we looked at the distribution of variable importance based on 100 model permutations (Figure 5). For the full time series model, of all input features, LPI was the most important, followed by PA and COHESION, with a mean accuracy decrease of approximately 0.18, 0.13, and 0.11, respectively. Similarly for the annual model, LPI was the most important variable, with a mean accuracy decrease of approximately 0.18, followed by PA (0.14) and AREA_CV (0.08). The least important variable for the full time series models was LSI (0.05) and COHESION for the annual model (0.03).

### Temporal evolution of infestation probabilities

**Fig. 6:**
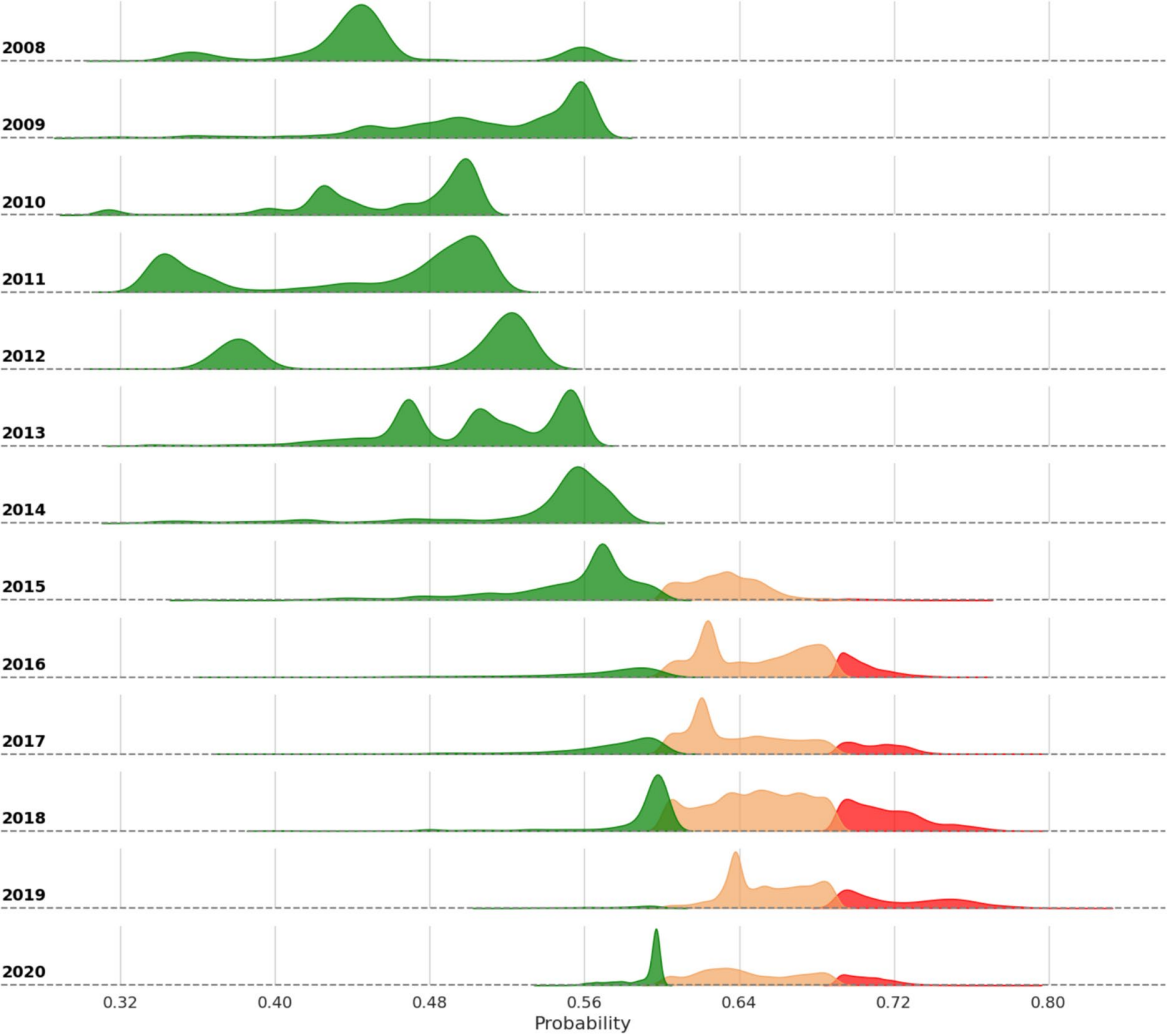
Illustration of the distribution of infestation probabilities between 2008 and 2020 for the entire LSJ region. The orange shade indicates infestation likelihood greater that 60%, while red indicates high infestation severity according to the AUC threshold (69%)

**Fig. 7:**
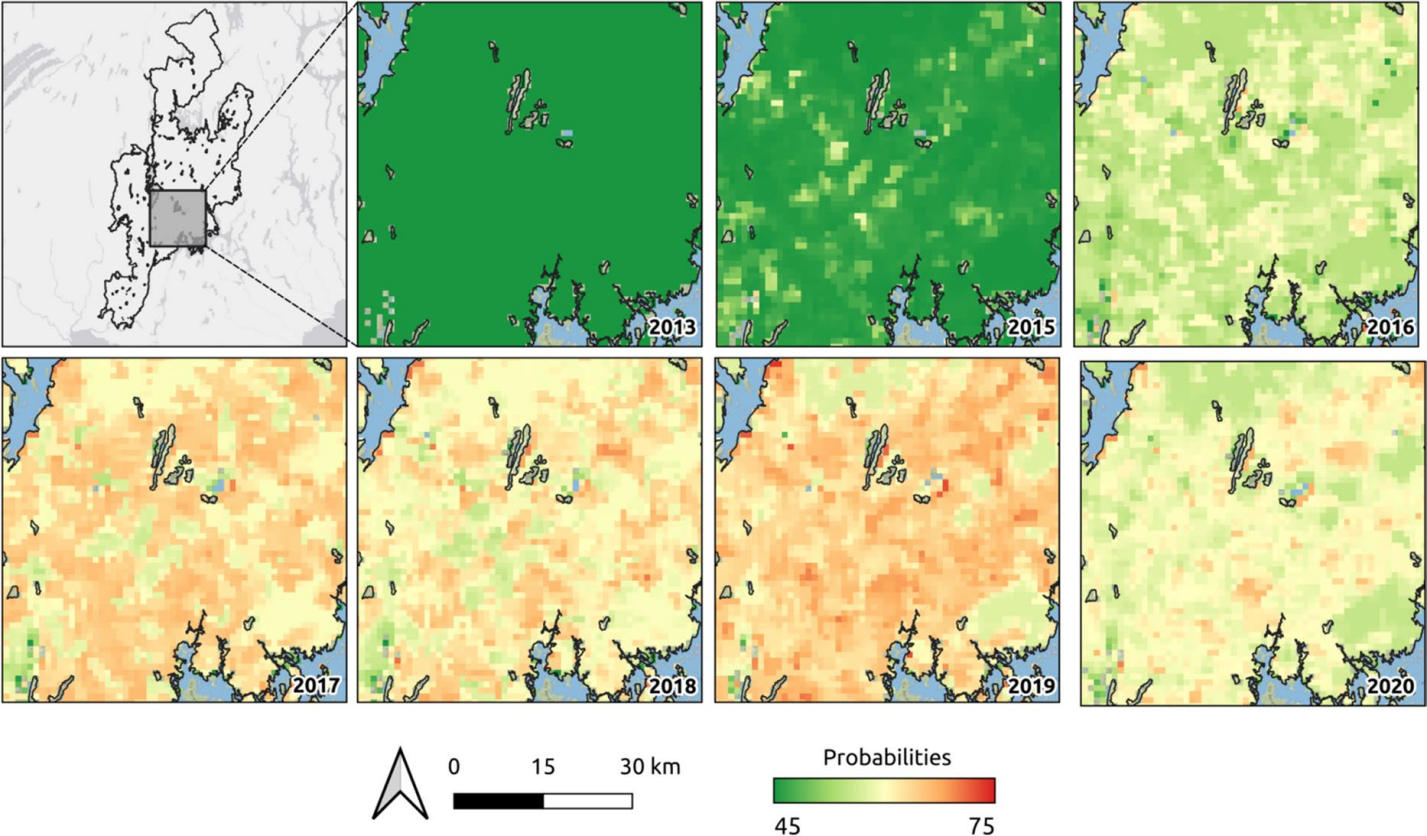
Panel illustrating the progression of infestation probabilities as extracted from the annual random forest models. Only selected years are displayed for better visualization. Probability values are expressed as percentage

**Fig. 8:**
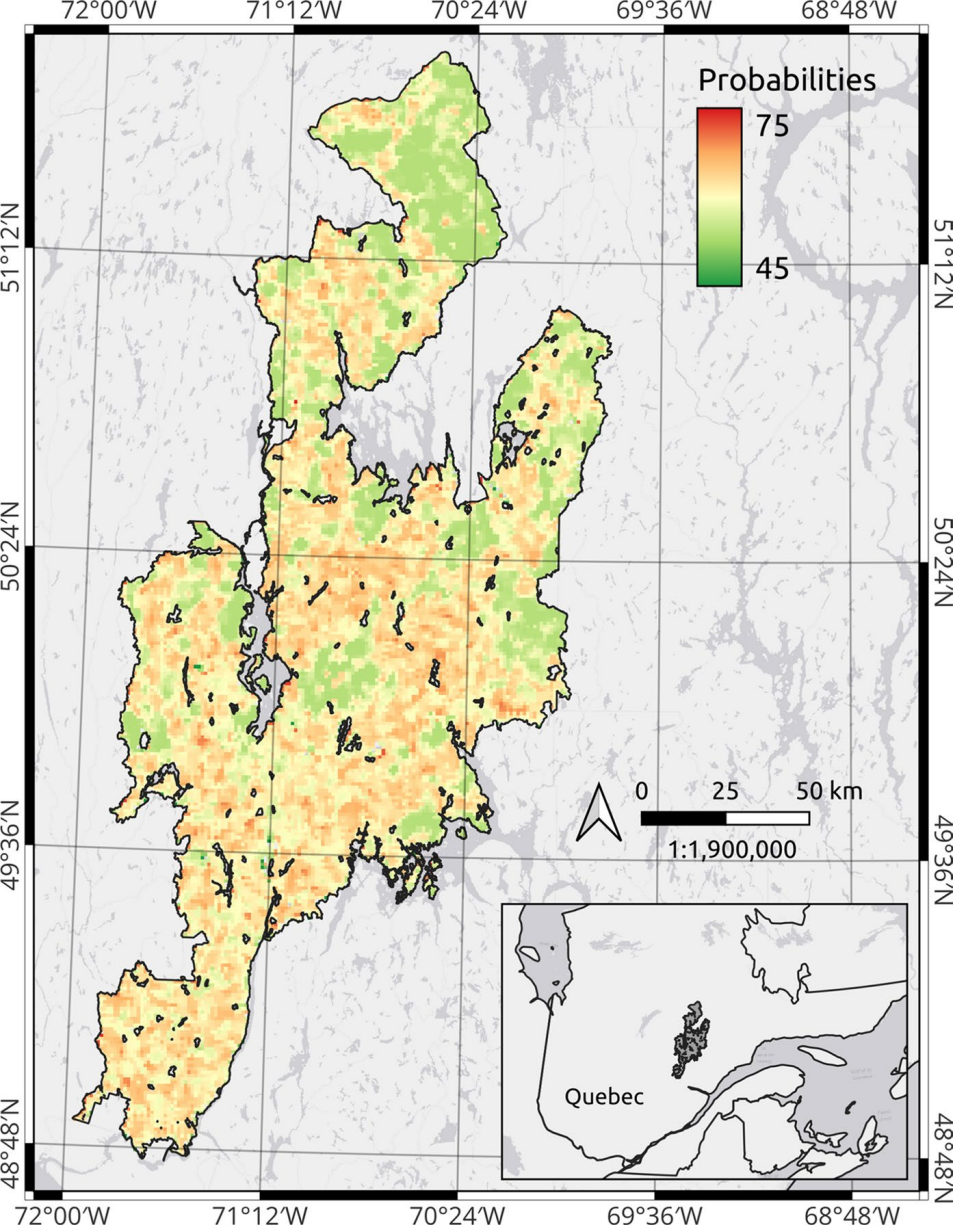
Map of infestation probabilities as estimated from the random forest model in 2019 for the entire LSJ. Probability values are expressed as percentage

We observed strong agreement between our infestation probabilities and the expansion of SBW based on the AOS surveys, on an annual basis across the entire region (Figure 6). The predicted infestation from the Landsat-derived stands was initially low in the 2008 model, with a mean probability of 45%, indicating low levels of attack on these initial stands. Modelled probability estimates increased in 2015, reaching a maximum in 2019 (mean 67%). From 2016 onward, most of south-central LSJ was estimated to have higher infestation probabilities than in previous years. A zoomed view of the temporal progression of the infestation probabilities is provided in Figure 7, while a complete map for LSJ in 2019 is shown in Figure 8.

## Discussion

### Fragmentation of conifer patches increases infestation likelihood

As fragmentation of conifer patches increased across the landscape, so did infestation severity. This suggests that (i) SBW infestations preferentially onset on landscapes with greater fragmentation of conifer patches, and that (ii) the model can identify landscape configurations that are more likely to undergo severe or moderate infestations. So, our approach not only models the risk of severe or moderate infestation associated with SBW movement across the landscape, but also the success of SBW within stands over multiple years. Moreover, we were able to capture areas where severe or moderate infestations were not yet occurring but may be likely to occur given the current landscape configuration. Thus, our model can provide more fine-grained information on SBW infestations than the AOS surveys, which are generally more suited for broad-scale assessments.

Increased likelihood of infestation in landscapes with fragmented conifer patches, where these are intermixed with other land cover types, may be associated with a variety of complementary mechanisms. One possible interpretation for increased severe or moderate defoliation under a more fragmented landscape is that it may be related to the cyclic phases of infestation. Specifically, populations grow by consuming available resources until unsustainable levels are reached, at which point the population and larval recruitment start to decline (Cooke et al. 2007; Régnière and Nealis 2019), as we observed lower likelihoods in 2020 (Figure 6). In landscapes where host species are more fragmented and resources are more limited, SBW individuals concentrate. As populations peak and reach epidemic levels, resources are consumed more avidly and defoliations become more severe. In parallel, we observed a consistent northward movement of SBW severe infestations in the study area. While SBW is omnipresent in these landscapes, where populations inhabit fir and spruce forests at endemic levels (Mattson et al. 1988), such movement may be a result of changes in landscape configuration and composition in more recent years, coupled with the ability of SBW adults to disperse over long ranges (Boulanger et al. 2017; Sturtevant et al., 2013). This movement of SBW adults allow populations to concentrate and saturate new portions of the landscape. Moreover, it may further increase the risk of severe or moderate defoliations to occur in stands not previously defoliated (Cooke 2022). Changes in landscape configuration that favor such landscape saturation, and thus the higher infestation risk, were modelled. We reported a loss in conifers throughout the study area, which may have resulted in the crowding of smaller patches of host species, effectively favoring the establishment of more severe infestations in those regions. Therefore, our approach was capable of modelling the risk of landscape infestation associated with SBW saturation of smaller patches due to increased conifer fragmentation.

We did not explicitly capture any information related to individual species composition within the coniferous land cover class, which may have resulted in mixed effects on mortality (Zhang et al. 2018, 2020; Kneeshaw et al. 2021). For example, the overall lower infestation risk in northern regions may also have been related to variability in species composition, whereby higher abundance of secondary host species such as black spruce may have reduced infestations likelihood in those areas simply because there may have been fewer balsam fir trees. Similarly, in the south, more intense management may have favored the establishment of balsam fir over other species (Bouchard et al. 2008; Kneeshaw et al., 2022). Therefore, species composition can be included as an explanatory variable to identify preferential sites for population concentration and to model how the risk of infestations changes for specific species combinations under different infestation severities. However, McNie et al. (2023) showed that the likelihood of infestation onset is more dependent on landscape configuration than it is on composition. Nevertheless, to reduce the influence of such latitudinal species composition gradient, we imposed a maximum search system for the estimation of infestation probabilities by including only tiles that were close to the epicenter of the infestation for each year in the random forest models. Through this approach, we were able to discriminate areas at a higher risk of infestation via potential saturation from those that were likely to remain undisturbed or remain at endemic population levels, while controlling for the northward movement of the attacks through the landscape. Moreover, by imposing a search radius, we limited the selection of tiles to those that were within the median dispersal range of migratory adults for a given year during severe infestations that began in the south (Royama 1984, 2005; Sturtevant et al. 2013).

### Diverse effects of configuration metrics on infestation probability

Greater model dependence was observed on metrics describing size and aggregation, suggesting that these are major control factors in determining the risk of severe or moderate infestations. However, their influence varied on an annual basis (Figure 4). One possible explanation is that severe SBW infestations generally take four or more years to kill host trees due to lagged growth reduction responses (MacLean 1980; Krause et al. 2012; Houndode et al. 2021). This suggests that mortality occurs following cumulative infestations. Thus, effects of landscape configuration on infestation severity may vary on an annual basis depending on the stage of the infestation and its severity (Bouchard & Auger 2014). Conversely, metrics describing shape complexity had comparatively lower influence on the model and were less important than size and aggregation metrics. Therefore, it seems that SBW infestations are affected more by area and connectedness of conifer patches than by their relative shape. However, PA and LPI varied greatly on an annual basis compared to LSI and AREA_CV. Therefore, these former metrics seem more robust indicators of SBW infestation likelihood when applied to time series data instead of annual observations.

Another possible explanation for the observed variability in the influence of landscape metrics on SBW infestation probability (Figure 4) and in the fitting of our annual random forest models (Figure 3) may be related to the greening up of disturbed forest patches (Spence and MacLean 2012). As infestation severity changes over time, it is possible that tiles that were originally disturbed in early years were not further disturbed until later years, effectively allowing stands to partially recover from past infestations. Under this scenario, annual models developed for later years may include stands with mixed disturbance histories. This is particularly important for the annual random forest models we built, and is more marginal in our full time series model, which combines information from all previous years. In the latter case, the same stand that was initially infested and had time to recover before another infestation was included in the model, effectively capturing its disturbance history. Nevertheless, we would expect landscape configuration not to be as impacted by regrowth of infested stands as other stand attributes such as species composition and vertical forest structure (Bouchard et al. 2006; Bouchard and Pothier 2010; Kneeshaw et al. 2022).

### Integrating remote sensing into landscape configuration research

Remote sensing offers opportunities to utilize wall-to-wall, spatially-explicit, open-access datasets and by-products at varying spatio-temporal scales (Hermosilla et al. 2016; Crowley and Cardille 2020; Wulder et al. 2022; Fassnacht et al. 2024). This includes the ability to map forest stands on an annual basis, in contrast to the decadal updates that the FRI is subject to (Wulder et al. 2024). The object-based stand segmentation routine we utilized was previously employed to generate a stand-level inventory for Canada’s forests from satellite imagery (Wulder et al. 2024). One strength of this approach lies in the use of reproducible, objective, and consistent input data and methods, which permitted us to investigate the temporal evolution of landscape configuration. Moreover, auxiliary data can be included as part of the segmentation in a similar manner to photointerpretation. While we included the year of fire and harvesting, elevation maps and structural layers, for example derived from lidar, can provide additional information on the landscape to improve the results of image segmentation (Morgan and Gergel 2010).

We implemented a tiling system to generate spatially-explicit estimates of infestation probabilities, which allowed us to identify areas more likely to experience severe and moderate infestations at annual time steps at a moderate spatial resolution by considering multi-scale contextual information. While our tiles were sufficiently large to encompass an average stand (16 ha), information from neighboring tiles enabled the extension of our probability prediction to include contextual information, effectively incorporating multi-stand dynamics. These have been shown to be important predictors for defoliators with similar ecology to SBW, such as western spruce budworm (*Choristoneura occidentalis* Freeman) in British Columbia, Canada (Senf et al. 2017a, b).

### Modelling limitations and variability in infestation dynamics

One limitation of our approach was the exclusion of species composition layers for model training purposes. While Hermosilla et al. (2022a, 2024) mapped and characterized dominant tree species distribution and dynamics across Canada, results on co-occurring or non-dominant species are more complex to interpret and require careful use (Hermosilla et al. 2024). Instead, we relied on more temporally stable land cover maps (Hermosilla et al. 2022b). However, we did not included information on land covers other than conifers in our models. These covered a small proportion of the study area, yet may be of interest to include in studies where mixedwood and deciduous forests are more predominant as these may influence the actual infestation severity for a given severity class as reported by the AOS. Further, we did not consider environmental variables influencing SBW population dynamics, such as temperature, precipitation, or elevation, which may affect the spatio-temporal variability of an infestation (Régnière et al. 2012; Bouchard and Auger 2014; Li et al. 2020). Additionally, while our model was successful at mapping the risk of SBW infestations across the landscape at annual time steps, our intent was not to predict future infestation probabilities. For example, it can be argued that stands that were not initially infested may have a greater probability of being infested in the future (Magnussen et al. 2004; Bouchard and Auger 2014; Senf et al. 2017a, b; Li et al. 2020; Nealis & Régnière 2021). Our research did not explicitly investigate this aspect. Our goal was to assign probability estimates based on the proximity of annual landscape configuration measurements to those that underwent some form of infestation during the analyzed period and within the defined search radius. Hence, we were solely interested in interrogating landscape configuration changes under SBW infestations. However, we developed a full time series model to include landscape configuration changes over 13 years. This approach allowed us to better capture multi-year SBW infestation dynamics in relation to landscape configurations. Future research focused on predicting future SBW infestation could integrate landscape configuration information as explanatory variables to assess the risk of future SBW infestations across the landscape.

In contrast to our results, studies have suggested that stands with greater stand connectivity can favor the expansion of insect infestations (Kneeshaw et al. 2021). Similarly, other studies have documented that the movement of insect infestations is contingent on high stand connectivity as well as high concentration of host species (Johnson et al. 2004). This highlights the fact that greater connectivity and host abundance may be requirements for endemic populations to escalate to epidemic levels (Hartl-Meier et al. 2017; Kneeshaw et al. 2021). However, studies agree that this may also depend on the phase of infestation (Bone et al. 2013). For example, Radeloff et al. (2000) concluded that the effect of landscape configuration varies for jack pine budworm populations (*Choristoneura pinus pinus* Freeman) depending on the phase of the outbreak. In particular, the authors documented that a greater density of jack pine edges correlated positively with jack pine budworm population densities during the peak of the infestation, and that this effect reversed as the outbreak declines. Bouchard & Auger (2014) found that the role of forest composition on SBW infestations was important during the short- and long-distance expansion phases. The authors documented greater movement of SBW populations in landscapes dominated by a greater proportion of suitable hosts. Other studies investigating SBW outbreak dynamics suggested that fragmented landscapes may increase the unpredictability of SBW infestatifons (McNie et al. 2024). Therefore, it seems that the transition to epidemic population may be governed by a myriad of factors, with effects depending on the location and phase of infestation (Bouchard and Auger 2014).

In our case, during the 13 year study period, SBW infestation gradually increased and dominated marked portions of the landscape. Therefore, we would expect the influence of forest configuration to vary over time and be particularly important as infestations onset and peak. We thus argue that the development of a combination of annual and full time series models, including the temporal variability of landscape configuration, would be beneficial to account for this underlying variability.

## Conclusions

In this research, we showed the importance of considering configuration, and not only composition, in understanding how and where SBW infestations are more likely to occur. Remote sensing data allowed us to produce annual spatially-explicit layers of SBW infestation likelihood as a function of stand area, aggregation, and shape metrics. Moreover, we included a set of complementary configuration metrics at varying scales to effectively capture multi-stand population dynamics. Ultimately, this work has the potential to be included in management strategies aimed at maintaining and improving forest resistance and resilience to SBW infestations.

## Supplementary Information

Below is the link to the electronic supplementary material.

Supplementary file1 (DOCX 1955 KB)

## Data Availability

The FRI data is available from the Ministère des Ressources naturelles et des Forêts du Québec at https://www.quebec.ca/agriculture-environnement-et-ressources-naturelles/forets/recherche-connaissances/inventaire-forestier/donnees-cartes-resultats. The disturbance layers derived from Landsat used in this paper are available at https://opendata.nfis.org/mapserver/nfis-change_eng.html.
