# Supplementary material for: Characterizing landscape configuration effects on eastern spruce budworm infestation dynamics
Source: Landsc Ecol. 2025 Aug 27;40(9):183. doi: 10.1007/s10980-025-02203-z (PMC12380986; doi:10.1007/s10980-025-02203-z)
Supplement: Supplementary file 1 — Supplementary file1 (DOCX 1955 KB) [file 10980_2025_2203_MOESM1_ESM.docx]

**Characterizing landscape configuration effects on eastern spruce budworm infestation dynamics**

Tommaso Trotto^1^*, Nicholas C. Coops^1^, Alexis Achim^2^, Sarah E. Gergel^3^, Dominik Roeser^1^

^1^Department of Forest Resources Management, University of British Columbia, 2424 Main Mall, Vancouver, BC V6T 1Z4, Canada

^2^Département des sciences du bois et de la forêt, Université Laval, 2425 rue de la Terrasse, Québec, QC G1V 0A6, Canada

^3^Department of Forest and Conservation Sciences, Faculty of Forestry, University of British Columbia, 2424 Main Mall, Vancouver, BC, V6T 1Z4, Canada

*Corresponding author: Email: ttrotto@mail.ubc.ca

ORCID:

Tommaso Trotto: 0009-0001-0307-7076

Nicholas C. Coops: 0000-0002-0151-9037

Alexis Achim: 0000-0003-0118-1651

Sarah E. Gergel: 0000-0003-2202-1403

Dominik Roeser: 0000-0002-8555-0903

***Fig. S1*** *Maps of change in landscape configuration metrics between 2013 and 2020. PA = patch area; LPI = landscape patch index (landscape percentage covered by the largest patch); COHESION = cohesion (patch connectedness); LSI = landscape shape index (shape complexity of a patch); AREA_CV = coefficient of variation of landscape area within a tile; SHAPE = shape (patch shape)*
